# Airborne virus transmission under different weather conditions

**DOI:** 10.1063/5.0082017

**Published:** 2022-01-13

**Authors:** Santosh K. Das, Jan-e Alam, Salvatore Plumari, Vincenzo Greco

**Affiliations:** 1 School of Physical Sciences, Indian Institute of Technology Goa, Ponda 403401, Goa, India; 2 Variable Energy Cyclotron Centre, 1/AF Bidhan Nagar, Kolkata 700064, India; 3 Homi Bhabha National Institute, Training School Complex, Mumbai 400085, India; 4 Department of Physics and Astronomy, University of Catania, Via S. Sofia 64, I-95125 Catania, Italy; 5 Laboratori Nazionali del Sud, INFN-LNS, Via S. Sofia 62, I-95123 Catania, Italy

## Abstract

The COVID19 infection is known to disseminate through droplets ejected by infected individuals during coughing, sneezing, speaking, and breathing. The spread of the infection and hence its menace depend on how the virus-loaded droplets evolve in space and time with changing environmental conditions. In view of this, we investigate the evolution of the droplets within the purview of the Brownian motion of the evaporating droplets in the air with varying weather conditions under the action of gravity. We track the movement of the droplets until either they gravitationally settle on the ground or evaporate to aerosols of size 2 *μ*m or less. Droplets with radii 2 *μ*m or less may continue to diffuse and remain suspended in the air for a long time. The effects of relative humidity and temperature on the evaporation are found to be significant. We note that under strong flowing conditions, droplets travel large distances. It is found that the bigger droplets fall on the ground due to the dominance of gravity over the diffusive force despite the loss of mass due to evaporation. The smaller evaporating droplets may not settle on the ground but remain suspended in the air due to the dominance of the diffusive force. The fate of the intermediate size droplets depends on the weather conditions and plays crucial roles in the spread of the infection. These environment dependent effects indicate that the maintenance of physical separation to evade the virus is not corroborated, making the use of face masks indispensable.

## I. INTRODUCTION

It is well known that droplets released by infected persons through coughing, sneezing, speaking, or breathing contain micro-organisms (bacteria, virus, fungi, etc.) causing a large number of diseases.^1,2^ Several decades ago, it was considered that the infections that are contained within the droplets are airborne.^3^ Recently, ten scientific reasons have been provided in support of SARS-COV-2 as an airborne infection^4^ (see also Ref. 5 for review). The transmission routes^6^ of the virus crucially depend on how the droplets evolve in space with time under the action of three competitive forces—gravitational force, which is opposed by the drag and diffusive forces. For example, big droplets settle gravitationally on different surfaces, such as handrails, door handles, and tables, and eventually infect through direct contact. Small droplets either directly discharged or formed due to evaporation or fragmentation of the big droplets remain suspended in the air for a long time due to the dominance of the diffusive force over the gravitational force, which infect healthy persons through inhalation. It still remains as a big challenge to understand the density of the SARS-COV-2 virus in the air,^7^ their capability to infect,^8^ their survivability on different surfaces,^9,10^ and the relative weight of these factors. Therefore, these factors limit our ability to evaluate the risk.^11^ As the saliva of the infected persons contains the Coronavirus,^12,13^ it can be transmitted through the process of respiration,^14,15^ speaking,^12,16,17^ coughing, and sneezing. The increase in emission of pathogens with the loudness in speaking, which may depend on some unknown physiological factors varying among individuals, has also been reported.^16^

Recently, it has been reported that the droplets and aerosols (droplets with size <5μm^18^) can travel a distance much larger than the prescribed six feet and remain suspended in the air for hours, which is supported by the fact that the SARS-COV-2 RNA is recovered in the air sample.^19^ The propagation and aerosolization of the droplets have been investigated within the purview of statistical mechanics and fluid dynamics.^20–22^ The Euler–Lagrange equation has been applied to study the effects of the size, ejection velocity, and angle of emission of the droplets on their trajectories. However, the application of the stochastic statistical mechanics^23,24^ is particularly crucial to study the motion of small droplets and aerosols for which the airborne transmission turns out to be very vital. The Brownian motion of the aerosols in the air-bath can be studied by solving the Langevin stochastic differential equation.^25^ In the Euler–Lagrange approach, the stochastic motion is not included.

Here, we investigate the spread of these ejected droplets in the neighborhood of the infected individual under varying weather conditions. The result will be useful in planning the preventive strategies at different climatic conditions. The droplets ejected interact with the molecules of the still/flowing air at temperature (*T*) and relative humidity (RH) in the presence of gravity. The interacting forces between the droplets and the air molecules are changing continuously as the molecules are changing their coordinates continuously with time. This makes the problem very complex and it becomes impossible to find an exact analytical solution. The spread of the droplets, however, will depend on these interactions. Under such circumstances, the air molecules can be regarded as forming a thermal bath where the droplets are executing Brownian motion with its changing mass due to evaporation. The interaction of the evaporating droplets with the bath can then be grouped into drag and diffusive forces quantified through the drag and diffusion coefficients. Therefore, there are three types of force acting on a droplet, which are: (i) drag and (ii) diffusive forces between the droplet and the air molecules and the (iii) Newtonian gravitational force on the droplets due to their non-zero mass. However, it is crucial to note that droplets are undergoing loss of mass due to evaporation and hence the gravitational force is changing with time too.

In the present work, we investigate the propagation of the virus-containing droplets subject to continuous evaporation by solving the Langevin stochastic differential equation of statistical mechanics^23,24^ coupled with the equation that governs the evaporation of the droplets. The Langevin equation is applicable in the present context as the mass of the droplets is much higher than the mass of oxygen and nitrogen molecules forming the bulk of the air.

It is crucial to mention here that the flow of the droplet in the air may either be laminar or turbulent depending on the situation. In the present work, this fact has been taken into consideration with appropriate parameterization Reynold’s number (Re), which appears through the drag force.^26^ The inclusion of all the forces mentioned above enables us to study the trajectories of droplets with a wide range of sizes. It will be interesting to study how the evaporating droplets evolve in space and time under the influence of gravitation, which will act to pull the droplet on the ground in contrast to the diffusive and drag forces, which will prevent it from falling on the ground. Big (hence massive) droplets are expected to settle gravitationally quickly, and the smaller one is expected to remain suspended in the air for a longer time. However, under evaporation, the droplets suffer continuous loss of mass, and consequently, a droplet, which will otherwise fall on the ground due to gravitation, may not do so but remain suspended as smaller droplets/aerosol or isolated virus for a longer time before complete decomposition.

The present work is a sequel of a previous publication.^25^ In contrast to the earlier work, here we include the process of evaporation of the droplets and their flow beyond the laminar region. This paper is organized as follows. In Sec. II, the solution of the Langevin equation coupled with the equation that governs the evaporation has been presented. Section III contains the results, and Sec. IV is devoted to summary and discussions.

## II. SOLVING THE LANGEVIN EQUATION IN THE PRESENCE OF EVAPORATION

The Langevin equation, governing the motion of the droplet of mass (*M*) in the still air in the presence of a gravitational field,^23^ is given by

$$\frac{dr_{i}}{dt}=v_{i},$$ (1)

$$M\frac{dv_{i}}{dt}=-\lambda v_{i}+\xi \left(t\right)+F^{G}.$$ (2)

In Eqs. (1) and (2), *dr*_*i*_ and *dv*_*i*_ are the shifts of the coordinate and velocity in each discrete time step *dt*, and *i*(=*x*, *y*, *z*) stands for the Cartesian components of the position and velocity vectors. *λ* in Eq. (2) is the drag coefficient, which will be fixed soon. The first term in the right-hand side of Eq. (2) represents the dissipative force, and the second term stands for the diffusive (stochastic) force. *ξ*(*t*) is also called noise due to its stochastic nature. We study the evolution with a white noise ansatz for *ξ*(*t*), i.e., ⟨***ξ***(*t*)⟩ = 0 and ⟨***ξ***(*t*)***ξ***(*t*′)⟩ = *κδ*(*t* − *t*′), where *κ* is the diffusion coefficient, which regulates *ξ*(*t*). White noise describes a fluctuating field without memory, whose correlations have an instantaneous decay called *δ* correlation. The third term in Eq. (2), *F*^*G*^, represents the gravitational force (= *Mg*, *g* = 9.8 m/s^2^) acting on a droplet of mass M, which changes with time due to the evaporation. The mass of the droplet is related to its diameter (*D*) as *M* = *πD*^3^*ρ*_*L*_/6 ion, where *ρ*_*L*_ is the density of the evaporating liquid.

The rate of decrease of the diameter *D* of a spherical liquid drop due to evaporation is given by^26,27^

$$\frac{dD}{dt}=-\frac{4M_{L}D_{v}}{D\rho_{L}RT_{f}}\Delta p\left(1+0.276\;{Re}^{1/2}Sc^{1/3}\right).$$ (3)

In Eq. (3), *M*_*L*_ (= 0.018 kg/mol) is the liquid, *D*_*v*_ is the diffusion coefficient of the vapor molecules in the saturated film around the droplets, *T*_*f*_ is the average temperature of the film formed around the droplets due to evaporation, *R* = 8.3144 J/(mol K) is the gas constant, and Re is Reynold’s number^28^ given by

$$Re=\frac{D\rho_{a}\;v}{\eta_{a}},$$ (4)

where *v* is the relative velocity of the droplet with respect to the surrounding air, *ρ*_*a*_ is the density, and *η*_*a*_ is the viscosity of the air at temperature *T*_*f*_. *Sc* is Schmidt’s number, given by

$$Sc=\frac{\eta_{a}}{\rho_{a}D_{v}}.$$ (5)

Δ*p* is the difference between the vapor pressure near the droplet and in the atmosphere, which acts as the driving force for the transport of vapor away from the droplet surface. Δ*p* can be related to the saturated vapor pressure at ambient temperature (*T*) and wet-bulb temperature (*T*_*w*_) as

$$\Delta p=p_{sat}-p=\gamma \left(T-T_{w}\right),$$ (6)

where *p*_*sat*_ is the vapor pressure near the surface of the droplet, *p* is the vapor pressure in the ambient air, and *γ* is approximately constant (∼67 pa/K). *T*_*w*_ can be expressed in terms of *T* and RH as

$$T_{w}=T-\left[\left(a_{0}+a_{1}T\right)+\left(b_{0}+b_{1}T\right)\text{RH}+\left(c_{0}+c_{1}T\right){\text{RH}}^{2}\right],$$ (7)

with *a*_0_ = 5.1055, *a*_1_ = 0.4295, *b*_0_ = −0.047 03, *b*_1_ = −0.005 951, *c*_0_ = −0.000 040 05, and *c*_1_ = 0.000 016 6; for further details, we refer to Ref. 26.

The trajectories of the droplets depend on the ejected velocity (initial) and on the flow velocity of the ambience. It is obvious that droplets in still and flowing air conditions [such as in an air conditioned (AC) room or open air] will follow different paths. The results for both the scenarios of still and flowing air are provided below. The air flow velocity has been taken into account through the Galilean transformation of the Langevin equation. Since the virus carrying droplets follow different trajectories in still and flowing air, the preventive strategies for indoor and outdoor conditions should take care of this fact. Here, we consider a velocity profile for the air flow as $u\left(x\right)=u_{0}\left(1-\frac{x}{x_{\max}}\right)$ with its upward and downward components as zero to serve this purpose, where *x* is the running coordinate and *u*_0_ is the peak value of *u*(*x*) at *x* = 0, which will be varied to check the sensitivity of the results. Here, *x*_max_ is the maximum value of *x*, which may be restricted to the size of an AC room in the indoor condition. We will also consider constant (no dependence on spatial coordinates) air flow velocity to calculate the distance followed by ejected droplets in the outdoor condition.

The value of the drag coefficients *λ* is estimated by using the following relation:

$$\lambda =\frac{1}{2}C_{D}\rho_{a}\;S\;v,$$ (8)

where *S* is the projected cross-sectional area of the spherical droplet of diameter *D*. The following expression is used for *C*_*D*_^29^ to extend the validity of the model beyond the region of laminar flow,

$$C_{D}=\frac{24}{R_{e}}+\frac{6}{1+\sqrt{R_{e}}}+0.4.$$ (9)

The diffusion coefficient is obtained by using the Einstein relation:^24^ *κ* = *k*_*B*_*Tλ*, where *k*_*B*_ = 1.38 × 10^−23^ J/K is the Boltzmann constant. It may be noted that for *C*_*D*_ = 24/Re, the well known Stokes law is recovered.

The initial spatial coordinate of the droplet is *x* = *y* = 0 and *z* = *H*_0_, where *H*_0_ is the height (1.7 m) at which the droplet is released (nose/mouth), i.e., (*x*, *y*, *z*) = (0, 0, 1.7 m) is the point of ejection. Equations (1)–(3) have been simultaneously solved numerically to obtain the trajectories of the droplets with the inputs mentioned above. We use the Monte-Carlo method to sample the initial phase-space distribution of the droplets.^25,30,31^ The calculation is done for different initial radii of droplets varying from 10 to 200 *μ*m^32^ and the ejection velocity *V*_0_ = 21 m/s.^33^ The initial velocity (at *t* = 0) is uniformly distributed in the *x* − *y* plane with *v*_*z*_ = 0. We use the Euler method at second order including the diffusive (stochastic) force. We have checked the convergence and stability of our solutions with respect to the time step using analytical solutions of the Langevin equation for simple configurations and then using the conditions relevant for the present work to get the trajectories of the droplets. This numerical technique has been used to reproduce available results in the literature [for example, the results are displayed in Fig. 11(a) of Ref. 29] under the same conditions.

The solutions of Eqs. (1) and (2) enable us to calculate the horizontal distance (*L*(*t*)), traveled by the droplets from the point of ejection as a function of time as $L\left(t\right)=\sqrt{x{\left(t\right)}^{2}+y{\left(t\right)}^{2}}$. Its maximum value of *L*(= *L*_max_) dictates the stationary distance that to be maintained between infected and healthy persons to prevent the virus. The solution of these equations can also be used to estimate the maximum time (*t*_max_) of suspension of the droplets. In Sec. III, results for both *L*_max_ and *t*_max_ have been presented.

## III. RESULTS

The virus carrying droplets are ejected with different sizes and initial velocity in the ambience in widely varying climatic conditions. Therefore, the results obtained by solving Eqs. (1)–(3) with different sizes of the droplets under different meteorological conditions are exhibited here to understand the prevention measures to be adopted to avoid the infection. We assume that the ejection velocity is 21 m/s (unless stated otherwise), which is close to its highest possible value found experimentally in Refs. 33 and 34. For given weather conditions, droplets with highest ejection velocity will travel maximum distance. Consequently, it will also decide the maximum social distance to be maintained to avoid the infection. The weather effects have been taken into consideration through temperature, relative humidity, and wind flow. The effects of evaporation and correction to drag force for flow beyond the laminar region have been included as mentioned above. The value of temperature is 20° wherever it is not stated explicitly.

**FIG. 1.**
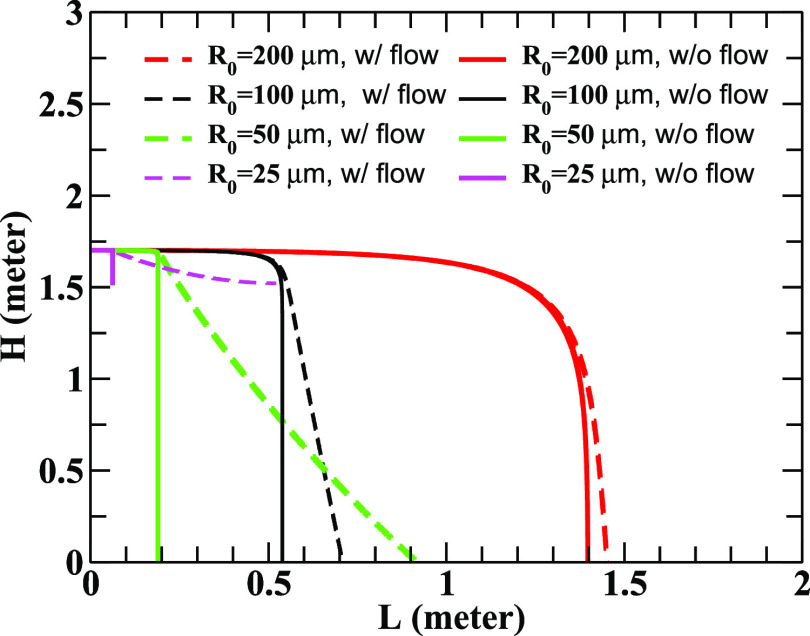
The variation of the height [*H*(*t*)] with the horizontal distance ($L\left(t\right)=\sqrt{x^{2}+y^{2}}$) is shown for droplets of various radii for the initial ejection velocity, *V*_0_ = 21 m/s, and the peak value of the wind flow velocity, *u*_0_ = 0.1 m/s. The results contain the effects of evaporation at ambience temperature 20 °C and RH 60%.

In Fig. 1, the variation of the height (*H*) with longitudinal distance (*L*) for droplets of different radii released at a height of 1.7 m is depicted. It is seen that a large droplet of radius 200 *μ*m propagates up to a distance of 1.4 m horizontally in the still air condition and 1.45 m in the wind flow condition with the peak velocity of the wind, *u*_0_ = 0.1 m/s. The interplay between the ejection velocity and the wind velocity can be understood from the following discussions. It may be mentioned here that the terminal or sedimentation velocity (*v*_*t*_) of a droplet of diameter *D* in air, obtained by balancing the drag plus the buoyant forces with the gravitational force, is given by

$$v_{t}=\sqrt{\frac{4}{3}\;g\frac{\rho_{L}-\rho_{a}}{\rho_{a}}\frac{D}{C_{D}}},$$ (10)

which reduces to the well known expression for *v*_*t*_ in the laminar flow region (with *C*_*D*_ = 24/*R*_*e*_) as

$$v_{t}=\sqrt{\frac{g}{18}\;R_{e}\frac{\rho_{L}-\rho_{a}}{\rho_{a}}}.$$ (11)

The effects of wind flow become insignificant if the terminal velocity of a droplet is larger than the wind flow velocity. Here, the wind velocity acts along the horizontal direction, whereas the sedimentation or terminal velocity acts along the downward vertical direction. If the terminal/sedimentation velocity dominates, then the resultant will be tilted toward the downward direction, which will force the droplet to settle on the ground. For a large droplet of size 200 *μ*m, *v*_*t*_($∼\sqrt{D}$) is larger than *u*_0_(= 0.1), which makes the effect of wind flow marginal as observed in the results displayed in Fig. 1. The mass dependence of the distance traveled by a droplet can be understood simply by ignoring the diffusive force and assuming the motion of the droplet in the *x* − *z* plane. In such situations, the trajectory of the droplet in still air is given by

$$z=z_{0}+g\frac{M^{2}}{\lambda^{2}}\ln\left(1-\frac{\lambda x}{Mv_{x}^{0}}\right)+x\frac{\lambda v_{z}^{0}+Mg}{\lambda v_{x}^{0}},$$ (12)

where *z*_0_ is the initial value of *z* and $v_{z}^{0}$ ($v_{x}^{0}$) is the initial value *z* (*x*) component of velocity. The maximum allowed value of *x*, *x*^max^, is determined by this solution as $x^{\max}=Mv_{x}^{0}/\lambda$. It is important to note here that *λ* ∼ *R* ∼ *M*^1/3^ for fixed density. Therefore, *x*^max^ ∼ *M*^2/3^ indicates that heavier droplets travel more distance than the lighter droplets for given $v_{x}^{0}$. This is supported by our numerical calculations (see also Ref. 35). The trajectory is terminated at *x*^max^ because at this point the forward momentum is completely spent to overcome the drag (frictional) force exerted by the air. A droplet of size 100 *μ*m travels a distance of 0.55 and 0.7 m, respectively, in still and wind flow conditions, implying that the wind flow has larger effects on smaller droplets as their terminal velocity is smaller. The effect of wind flow is noticeable for 50 *µ*m droplets. We observe that the droplets of size 25 *μ*m evaporate to aerosols before settling on the ground. The diffusive force dominates over the gravitational force for small droplets, which enable them to float in the air for a longer time (see also Ref. 36). For higher wind speed, the effects of the terminal velocity are small, for example, a droplet of radius 100 *μ*m can travel about 7.4 m for a constant wind speed of 2 m/s as seen below (see also Ref. 37).

**FIG. 2.**
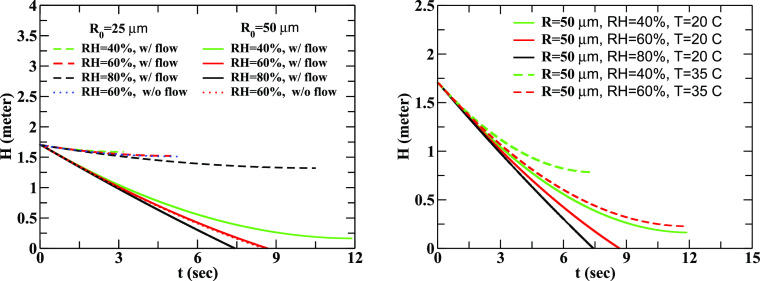
Left panel: the change in the height, *H*(*t*), as a function of time for droplets of different sizes has been depicted. Here, the initial ejection velocity, *V*_0_ = 21 m/s, and the peak value of the wind flow velocity, *u*_0_ = 0.1 m/s, at *T* = 20 °C. Right panel: same as the left panel showing the sensitivity of results on the ambient temperature (*T* = 20 and 35 °C). The results are derived with the inclusion of evaporation.

Figure 2 illustrates the time that droplets of different sizes take to settle gravitationally under different conditions of relative humidity and wind flow. We find that the droplets at smaller RH evaporate and do not settle on the ground due to weaker effects of gravity. For larger values of RH the evaporation process is slowed down, resulting in smaller loss of mass and hence forcing it to fall on the ground under the action of gravitation. Comparison of results for droplets of initial radius 50 *μ*m for RH = 40%, 60%, and 80% indicates that the effects of RH are significant. The droplets with initial radius 25 *μ*m or less, however, remain suspended in the air, namely, these droplets do not settle under the action of gravitation but continue to diffuse in the air making the use of masks mandatory to prevent the infection. It needs to be reiterated at this point that we follow the trajectories of droplets of radius up to 2 *μ*m created by the process of evaporation. At higher temperatures, the evaporation becomes faster as can be seen from the results displayed in Fig. 2. A droplet of initial radius 50 *μ*m evaporates more swiftly at 35  °C than at 20  °C at the same relative humidity of 40%. It is observed that at higher RH and low *T*, the effect of evaporation is small and hence the droplets survive for a longer time. The relation between the climatic condition and index of airborne infection rate and concentration rate of particles in saliva can be found in Ref. 38 (see also Ref. 39).

**FIG. 3.**
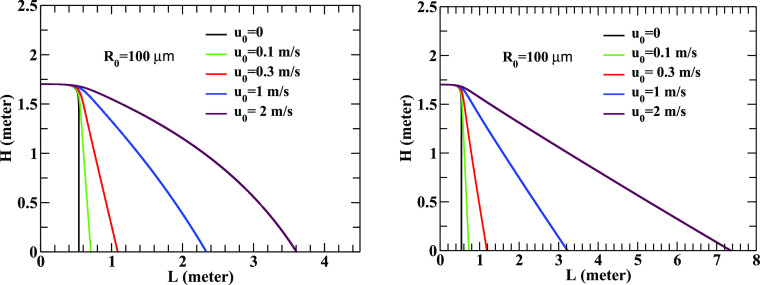
Left panel: the change in the height, *H*, as a function of *L* for a droplet of radius 100 *μ*m for different peak values (*u*_0_) of the wind velocity profile at *T* = 20° and RH = 60%. The droplet is ejected in a room of size 5 m with the initial ejection velocity of 21 m/s. Right panel: same as the left panel for different wind velocities of constant values (independent of space coordinates) as indicated in the figure.

The effects of wind flow in indoor (left panel) and outdoor (right panel) conditions are demonstrated in Fig. 3. The left panel shows the results for the flow profile mentioned above for various values of *u*_0_ in a room of size 5 m (which sets the value of *x*_max_). The right panel indicates the results for different wind flows with constant speed (independent of space coordinates) in an open air, say. It is clearly observed that the wind flow substantially affects the distance traveled by the droplets. We find that a droplet of radius 100 *μ*m ejected with a velocity of 21 m/s traverses a distance of 3.6 m (for the velocity profile mentioned above with *u*_0_ = 2 m/s) and as large as 7.4 m for a constant velocity of magnitude of 2 m/s at *T* = 20  °C and RH = 60%. This indicates that the droplet released by an infected individual can travel distances much larger than prescribed 2 m if the wind velocity is large.

**FIG. 4.**
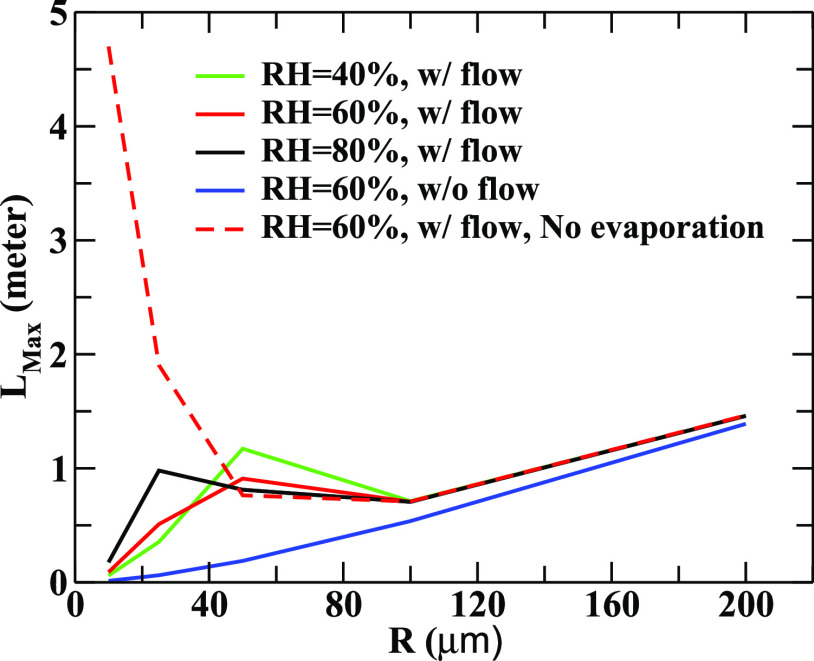
The variation of the maximum horizontal distance (*L*_max_) traveled by the droplets as a function of the droplet radius for different relative humidity values is shown. Here, initial ejection velocity *V*_0_ = 21 m/s and the peak value of the wind flow velocity *u*_0_ = 0.1 m/s.

In Fig. 4, we have displayed the variation of the maximum horizontal distance traveled by droplets as a function of radius at different RH values. The droplets of small and intermediate sizes are strongly influenced by the RH. We notice mild effects of flow (for small *u*_0_) and RH on bigger droplets. However, the droplets with intermediate radii are influenced more due to variation in RH and flow mainly due to their longer lifetime (from ejection to the formation of aerosol of size 2 *μ*m) in the air than the smaller droplets, which are subjected to quick evaporation.

**FIG. 5.**
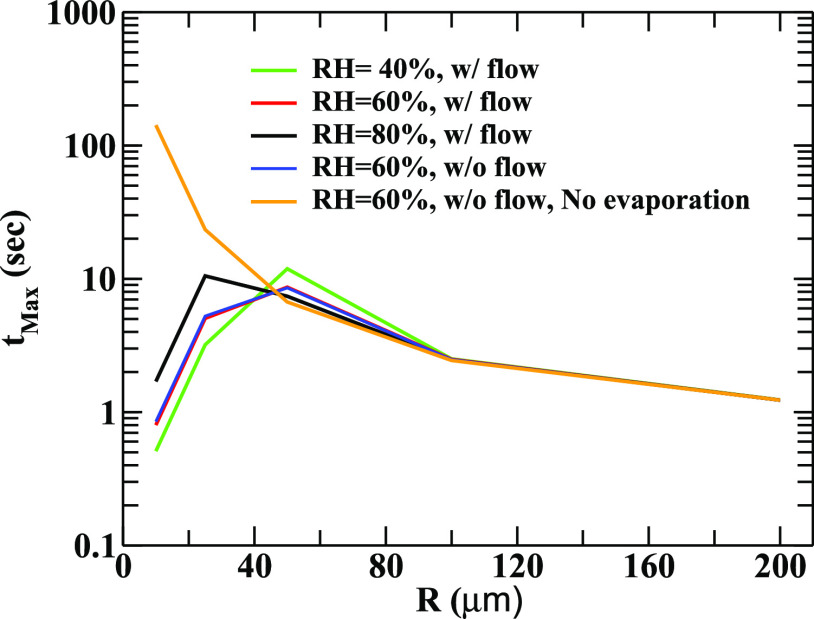
The variation of the maximum time the droplets take to settle on the ground under the action of gravity and the time the smaller droplets take to evaporate to generate aerosols of average radii 2 *μ*m are displayed here. The sensitivity of the results on the relative humidity has also been shown for *u*_0_ = 0.1 m/s.

Now, we would like to estimate the maximum time of suspension of the evaporating droplets of different radii in the air before settling gravitationally at different weather conditions. Relevant results to address this issue are displayed in Fig. 5. The effect of evaporation is found to be significant for small droplets (R < 40 micrometer). At higher RH, the droplets have the higher survival probability due to lesser mass loss by evaporation, and hence, they travel larger distance in the air. However, the time of suspension of intermediate and smaller sized droplets is very sensitive to the evaporation. Through the process of evaporation, smaller droplets may generate isolated virus, which may survive for more than an hour.^40^ However, droplets having larger radius fall on the ground quickly (smaller *t*_max_) due to stronger gravitational force compared to the diffusive force.

**FIG. 6.**
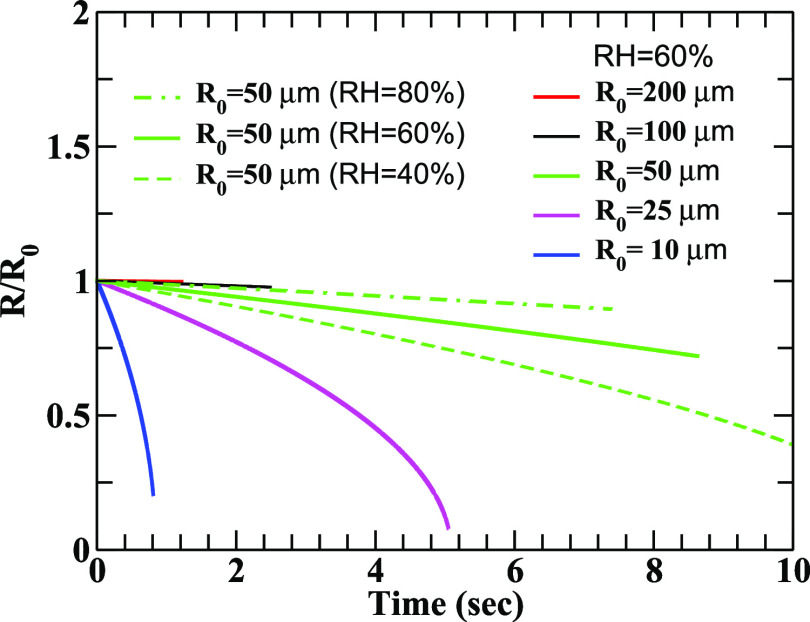
Change in the running radius *R*(*t*) normalized to the initial radius is depicted as a function of time for different relative humidity values. Here, *R*_0_ is the initial radius of the droplets. The values of *V*_0_ and *u*_0_ are taken as 21 m/s and 0.1 m/s, respectively.

**FIG. 7.**
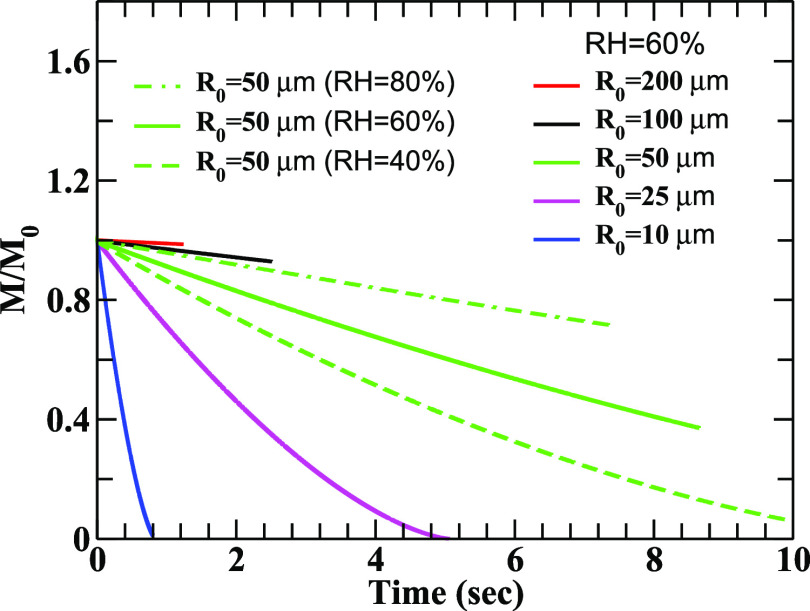
Same as Fig. 6 for running mass to initial mass of the droplets.

The sensitivity of evaporation on RH is clearly demonstrated through the results depicted in Figs. 6 and 7. In Fig. 6, we have plotted the change in the running radius (*R*) of the droplets normalized to their initial values (*R*_0_) for different humidity values. The reduction of mass due to evaporation is lower at higher RH, which allows the droplet to survive longer. It is noticed that smaller droplets are very sensitive to evaporation and they generate droplet nuclei before reaching the ground. In Fig. 7, the change in the running masses (M) of the droplet normalized to their initial masses (*M*_0_) is illustrated as a function of time for different humidity values. The results are consistent with those displayed in Fig. 6. The results clearly indicate the effects of RH on the evolution of the droplets, for example, a droplet of radius 50 *μ*m will approximately loss 60% and 20% of its mass due to evaporation at RH = 60% and 80%, respectively.

## IV. SUMMARY AND DISCUSSIONS

We have investigated the evolution of the droplets ejected during coughing, sneezing, or speaking by solving the Langevin stochastic differential equation with the inclusion of the evaporation process. The drag, diffusive, and gravitational forces are included in this study. The correction to the drag force in the non-laminar (turbulent) flow region implied by a large Reynolds number has been implemented here by using the parameterization of the Reynold’s number dependence of the drag coefficients. The droplets of various sizes have been considered with high ejection velocity (21 m/s) to get the upper limit of the distance travel by the droplets. The effects of different weather conditions have been taken into consideration through temperature, relative humidity, and wind flow. It is found that the maximum distance that a large droplet of size 200 *μ*m travels is about 1.5 m (*u*_0_ = 0.1 m/s), which is not affected significantly by low wind velocity. The tinier droplets diffuse through the air for a long duration^41^ due to weaker gravitational influence.

An infected person emits droplets of varying sizes. In addition to direct emission, smaller droplets can be created by the mechanism of evaporation (see also Ref. 42) and fragmentation.^43^ It is found that droplets of smaller size evaporate to create aerosols, smaller the sizes quicker they evaporate. Isolated virus generated by the process of evaporation can survive in the air for more than an hour.^40^ The evolution of these droplets will be controlled by the diffusive forces, and such droplets may remain suspended in the air for hours. However, it has also been reported^44^ that a multi-phase turbulent gas cloud is produced along with the droplets at the time of coughing and sneezing. The droplets within the envelope of this gas cloud may evade evaporation and prolong their survivability as isolated droplets. Droplets of larger size (say, 100 *μ*m) can travel a distance as large as 3.6 m in the indoor condition (for the flow profile defined above) and even more in the outdoor condition (i.e., constant flow velocity) if the wind flow is strong. It is appropriate to mention at this juncture that the intermediate size droplets are very sensitive to the weather conditions. The climatic conditions determine whether the intermediate size droplets will settle in the ground under the action of gravity or evaporate to aerosol. This indicates that the weather conditions play a crucial role in deciding the severity of spreading of the infection. Therefore, the mask and face shield should be used to prevent the virus.^45–47^ The maintenance of ventilation in indoor situation is also very crucial to avoid the infection.^48,49^

The maintenance of only a social distance of 2 m as a norm to avoid the virus is not corroborated by the present investigation. The aerosol created by the evaporation may remain suspended in the air for a long duration, and under strong wind flow, the droplets can travel a distance much larger than 2 m. The trajectories of the small droplets/aerosols are determined by the diffusive force as it dominates over the gravitational force. These trajectories are highly zig-zag in nature, and therefore, the path length traversed may be large but their mean values are small and such droplets remain suspended for a long time. The calculations based on Wells–Riley probability and computational fluid dynamics^50^ suggest that the use of masks in a classroom environment will be more effective than maintaining the physical distancing to escape the virus. In two Wuhan hospitals, the micrometer and sub-micrometer droplets of Sars-COV-2 were found at a distance of about 3 m away from the bed of an infected person.^51^

## Data Availability

The data that support the findings of this study are available within the article.
